# Medical and Physical Intelligence

**Published:** 1802-06

**Authors:** 


### MEDICAL AND PHYSICAL INTELLIGENCE.

[ FOREIGN AND DOMESTIC. ]

### Of the Movements of odorous Substances, placed on the Surface of Water.

Citizen Benedict Prevost has made several curious inquiries concerning the movements of odorous substances placed on the surface of water, of which the following is an extract. It is a fact long known to Naturalists, that small pieces of camphor, placed on water, move round, and turn with the greatest rapidity; a phænomenon which has also been observed by Messrs. Volta and Brugnatelli to take place with benzoic and succinic acid. Cit. Benedict Prevost, however, has shown, that a great number of different odoriferous substances possess this remarkable property. But though the fact itself is sufficiently ascertained, Naturalists differ very much in the explanations they respectively give of this curious phænomenon. Cit. B. P. attributes these movements to the emanation of the odorous particles; an opinion which he has built on numerous experiments. Mr. Venturi, however, professor of natural philosophy at the university of Modena, applies to these phænomena the explanation which is given by Cit. Monge, of the seeming attractions of bodies that are floating on the surface of water; and, according to him, the water has more attraction for the solid camphor than for the little portion that is dissolved in it, and with which it is saturated; it rises along the solid piece, describing there a curvelined inclined surface, along which that little dissolved portion descends, and in coming down repels, according to the mechanic laws, both the surface itself and the solid body to which it adheres. This effect however, he thinks, ought not to be be miſtaken for the repulſions which the air, imbibed by ether, or the hot exhalations of camphor, produce on light bodies that are floating on the ſurface of water, as in this caſe the preſence and influence of an elaſtic fluid appears evident. Doctor Corradori is of a different opinion, and he endeavours to explain this movement by an elective affinity of a kind of oil, which he ſuppoſes to proceed from the camphor at its contact with water. He thinks, that the repulſion of the water which takes place when we put camphor or other odorous ſubſtances on a china plate, or on wet glaſs, is effected by the elective attraction of the ſurface of the plate or glaſs for the oil emitted by thoſe ſubſtances, and, as he imagines, it is the oil which repels the water, and takes the place of it. For ſupporting his opinion, Dr. Corradori aſſerts, that camphor does not move on water, if it is incloſed in very ſmall veſſels, becauſe he never was able to move ſmall plates of metal, by placing on them pieces of camphor, and letting them float on water; an experiment which, however, proves ſuccefsful, when proper care is taken, and the utmoſt accuracy employed.

Cit. Prevoſt has replied to Dr. Corradori, in a memoir addreſſed to the *Société Philomatique* at Paris, which is entitled, "*Nouvelles Experiences sur les Mouvemens spontanés de diverses Substances à l'approche ou in contact les unes des autres.*" The principal facts contained in this paper are the following: A drop of ether placed on a plate of tin, of the weight of 13 grammes (about 3 ¼ drachms) made the water move with velocity, though it did not touch the ſurface of it. Thus the ether acts on the water at a diſtance; a fact, which may be proved in a very ſimple manner: A ſmall plate of tin placed on water, will give way on approaching it at a diſtance of ſome centimetres with the end of a glaſs tube moiſtened with ether. Small pieces of camphor thrown on well dried quickſilver will experience the ſame movements as water; but for making this experiment ſucceed, it is neceſſary to clear and dry the quickſilver as much as poſſible, as the ſmalleſt particle of oil or greaſe that is left on its ſurface will ſtop the movements. The pieces of camphor, however, muſt be very minute, the reaſon of which will be afterwards explained. Very minute ſcales of mica, loaden with a ſmall piece of camphor, and placed on quickſilver, move as on water. The benzoic acid turns likewiſe on quickſilver, but it muſt previouſly be reduced to extremely minute particles: an areola of oil forms itſelf around them when they are placed on the ſurface of water, which phænomenon is never to be perceived in camphor, even by means of a microſcope; but the metallic glaſs of the quickſilver is not changed by it. It reſults from theſe facts, that the preſence of water is not always required for theſe movements. As the movements will proceed on plates of alum, gum arabic, and of earthen ware, as well as on a moiſtened china plate, it appears, that no particular elective attraction of the oily or odorous ſubſtance to the plate takes place. The camphor likewiſe moves in very narrow veſſels, notwithſtanding the above aſſertion of Dr. Corradori; for Cit. Prevoſt has ſeen it move and turn turn in capillary tubes, into which it was brought in extremely minute particles. Cit. Prevoft is inclined to admit in thefe phenomena an intervention of an elaftic fluid. To thefe obfervations Cit. Biot makes the following additions, by which the queftion concerning the movement of the camphor on water may be decided. When we cut a fmall piece of camphor in the fhape of a cone, and hold the point of it towards a fmall piece of gold leaf that floats on water, this will be repulfed, as foon as we bring the point of the cone to it, within the fpace of 4 or 5 millimetres, in which way we may drive it all over the furface of the water without ever being able to touch it: the water, however, muft be very pure, and the veffel perfectly clean. We may hold the camphor with a pair of pincers, or put it into the end of a glafs tube; but it muft have the fhape of a fmall cone, as a larger piece of an irregular *figure* would involve the light body into its atmofphere, and thus prevent its eafy movement. The fame effects are produced, on taking, in place of the camphor, a fmall piece of fine fpunge impregnated with camphorated water, or a glafs tube, moiftened at its end with a few drops of the fame folution.

When we cover a china plate with a thin ftratum of water, and apply to it at a diftance of a few millimetres, the point of the above cone, fo that the axis of the cone be perpendicular to the furface of that ftratum, the water will give way beneath the cone, and form a concentric circle with it, the interior of which is coloured with rainbow coloured rays, proceeding from the continuation of the axis, and extending themfelves from within towards without with a rapid movement. A few moments after, the circle begins to difcolour itfelf from the centre towards the circumference, and the iris difappears whether we continue to hold the camphor above the furface of the water or not: this phenomenon has been obferved at a temperature of 15° Reaumur. On throwing a fmall piece of fine fponge impregnated with ether on water, it begins to move as camphor, and a hiffing found is heard, fimilar to that when water is evaporated on a hot iron. On looking horizontally at the furface of water, we may perceive fmall fparkling particles proceed from the fponge, which extend themfelves on the water in a ferpentine direction at the diftance of fome centimetres, producing their iriffes, fimilar to thofe of the preceding experiment; but they difappear fhortly. During this emiffion, the fponge has a progreffive and a rotatory motion, which are evidently owing to thofe fparkling particles, to the impulfion of which it conftantly yields. The two firft of thefe experiments inform us, that the camphor acts on water at a diftance, and without touching it, and the third enables us to perceive the manner in which thefe movements proceed on that fluid.

From thefe facts we may now certainly draw the following conclufions.

Camphor moves on water by means of the emiffion of its component particles, an emiffion which becomes perceptible to our fenfes by the odour that attends it, and by the repulfions which it exerts exerts against the small light bodies that are floating on water.— This emission proceeds at all points of the surface of camphor, but more rapidly where it is on the same level with water; the centre of the camphor has a progressive motion, but the body itself a rotary motion. As the figure of the piece of camphor is changing every moment, the movement of its centre of gravity is neither uniform nor rectilinear, but varies continually. The quickness with which the camphor moves is greater the smaller it is in size, and consequently the motion increases, or is accelerated in proportion as the camphor evaporates. In the second part of his memoir, Cit. Prevost relates a great number of experiments made with inodorous substances, which produce on moistened glass the same appearances as odorous substances, oily or volatile. When we spread on a china plate moistened with a thin stratum of water, a small piece of wet fine linen, the water seems to be repelled around it, and to form a great quantity of rainbow coloured rays. These phenomena will be produced with all substances, animal as well as vegetable, with all sorts of liquors and saline solutions, and they do not only take place on a china plate, but also on plates of gum arabic, alum, &c.

Cit. Prevost concludes from these experiments, and several others, analagous to them:

1. That all liquors have the property of repulsing each other.

2. That all dry organised substances, or those that preserve a particle of organisation, emit on absorbing water an elastic fluid, which attracting part of the water, repels that which is around it on a moistened glass pannel. The first conclusion, however, seems to be contrary to the general law of the mutual attraction of the atoms of matter with respect to the hypothesis of an elastic fluid; it ought to be suggested, that before we have recourse to ascribing these phenomena to new causes, we should endeavour to explain them according to those which we already know, and distinguish the effects produced by odorous substances, from those occasioned by inodorous substances; in which manner we may, with greater probability, account for the repulsion of fluids by linen or paper, &c.; and this repulsion, perhaps, arises from the water running down the inclined surface which those substances have made around themselves on imbibing the fluid.

---

*On the true Difference between the Crocodiles of the Ancient and of the New World, by Citizen* CUVIER.

Naturalists have hitherto extremely disagreed with respect to the number and character of the species of crocodiles, and the synonymous terms, as well as the different figures, have been extremely confounded; and notwithstanding the variety of opinions on this subject, truth had escaped their researches. Cit. Cuvier, however, having examined more than sixty crocodiles of all sizes, found they may be reduced to two species, the character of each of which he determines in the following manner.

*Crocodile* with an oblong snout, whose upper mandible is cut out on on each ſide, to give room to the fourth tooth of the lower jaw; the hind feet are intirely palmated or web-footed.

*Caïman* with an obtuſe ſnout, whoſe upper mandible receives the fourth tooth into a particular hollow, where it lies concealed; the hind feet are but half palmated.

The firſt ſpecies occurs only on the continent of the ancient world, but the ſecond is found in the new world.

---

The Surgeons of the Fleet have generouſly preſented their friend and Phyſician, Dr. Trotter, with a piece of elegant plate, to the value of one hundred and ten guineas, in remembrance of the manly and liberal ſentiments he has expreſſed in favour of this neglected body of officers. The following is the inſcription.

Doctori THOMÆ TROTTER;

Claſſis Regiæ Medico Primario;

Multa de Patria bene merenti;

Hoc Munuſculum,

Pignus obſervantiæ ſummæ,

Chirurgi Navales

Lubentiſſime offerunt:

Et publicæ ſalutis, et privatæ amicitiæ,

Teſtimonium ſacrum eſſe

Voluere.

1802.

---

A COURSE OF CLINICAL LECTURES on the Principles and Practice of Medicine, will commence early in October next; taken from caſes of the patients of the Finſbury Diſpenſary in St. John's Square. By *Thomas Jameson*, M. D. Member of the College of Phyſicians of London, and of Edinburgh, and one of the Phyſicians to the Finſbury Diſpenſary. Three Lectures will be delivered every week between the hours of eleven and twelve o'clock at noon, for a term of three months; and the patients of the Diſpenſary will alſo be examined from twelve to two o'clock on the ſame days. Students will have an opportunity, which is ſeldom to be met with, of obſerving diſeaſes from their commencement to their termination. To accommodate Hoſpital Students, the terms will be three guineas for the Lectures, and five guineas to thoſe who attend both the Lectures and Diſpenſary practice.

